# Automated Micro-Object Detection for Mobile Diagnostics Using Lens-Free Imaging Technology

**DOI:** 10.3390/diagnostics6020017

**Published:** 2016-05-05

**Authors:** Mohendra Roy, Dongmin Seo, Sangwoo Oh, Yeonghun Chae, Myung-Hyun Nam, Sungkyu Seo

**Affiliations:** 1 Department of Electronics and Information Engineering, Korea University, Sejong 30019, Korea; mohendra.roy@gmail.com (M.R.); ehdals20907@korea.ac.kr (D.S.); hope2ryan@gmail.com (S.O.); 2 Department of Physics, Rajiv Gandhi University, Arunachal Pradesh, Doimukh 791112, India; 3 Maritime Safety Research Division, Korea Research Institute of Ships and Ocean Engineering, Daejeon 34103, Korea; 4 Department of Big Data Science, University of Science and Technology, Daejeon 305350, Korea; proin@proinlab.com; 5 Department of Laboratory Medicine, Korea University Ansan Hospital, Ansan 15355, Korea; yuret@korea.ac.kr

**Keywords:** lens-free, algorithm, telemedicine, cytometer, RBC

## Abstract

Lens-free imaging technology has been extensively used recently for microparticle and biological cell analysis because of its high throughput, low cost, and simple and compact arrangement. However, this technology still lacks a dedicated and automated detection system. In this paper, we describe a custom-developed automated micro-object detection method for a lens-free imaging system. In our previous work (Roy *et al.*), we developed a lens-free imaging system using low-cost components. This system was used to generate and capture the diffraction patterns of micro-objects and a global threshold was used to locate the diffraction patterns. In this work we used the same setup to develop an improved automated detection and analysis algorithm based on adaptive threshold and clustering of signals. For this purpose images from the lens-free system were then used to understand the features and characteristics of the diffraction patterns of several types of samples. On the basis of this information, we custom-developed an automated algorithm for the lens-free imaging system. Next, all the lens-free images were processed using this custom-developed automated algorithm. The performance of this approach was evaluated by comparing the counting results with standard optical microscope results. We evaluated the counting results for polystyrene microbeads, red blood cells, HepG2, HeLa, and MCF7 cells lines. The comparison shows good agreement between the systems, with a correlation coefficient of 0.91 and linearity slope of 0.877. We also evaluated the automated size profiles of the microparticle samples. This Wi-Fi-enabled lens-free imaging system, along with the dedicated software, possesses great potential for telemedicine applications in resource-limited settings.

## 1. Introduction

Analysis of micro-objects, e.g., cells and micro-particles, is among the major tasks in pathology, biological research, and material science research. Concentrations and other physiological information regarding cells, such as their size and shape, are crucial information for a pathologist or a physician to reach a diagnostic conclusion. For example, information about the red blood cell (RBC) concentration, white blood cell concentration, and platelet concentration of a patient’s whole blood sample plays an important role in early diagnosis of many diseases. In most labs, especially in resource-limited settings, this type of study is generally conducted using a conventional optical microscope and hemocytometer. In this conventional method, an expert manually inspects the samples, which is tedious and prone to subjective error. On the other hand, recent biomedical research frequently requires the analysis of increasingly large numbers of cell and particle samples [1]. Resource-rich laboratories often use sophisticated automated alternatives, such as a Coulter counter and flow cytometer, to handle large sample numbers [2]. However, these automated systems are bulky and expensive, which limits their application and makes them impractical for resource-limited settings. Furthermore, there is increasing demand for a compact, high-throughput, fast, and cost-effective point-of-care cytometry system that can be operated by a non-expert user. These are the major challenges that most of the biomedical labs are currently trying to address. Many research groups have made extensive progress in this direction. Current advances in micro- and nanotechnology have improved the utility of micro- and nanoscale devices and the possibility of overall device miniaturization. These miniature devices retain advantages such as low power consumption and the possibility of using batch processing to lower the unit cost. Kumar *et al.* recently demonstrated a simple, cost-effective way to detect sickle RBC disease [3]. Richards *et al.* used advanced microfabrication technology to demonstrate a novel micro-Coulter counter that can efficiently detect microparticles [4]. Stewart and Pyayt demonstrated a microscale flow cytometry system that uses advanced microfluidic technology to efficiently detect cells and their sizes [5]. All these methods are based on microfluidic technology and supported by an independent liquid flow source such as a peristaltic pump. However, these support systems are bulky and very challenging to miniaturize, which affects the overall size of the system. Many research groups successfully demonstrated other alternatives to detect and characterize microparticles. Among them, the imaging of microparticles and cells using a compact lens-free imaging system promises significant advantages [6]. Bishara *et al.* successfully demonstrated a compact lens-free holographic microscopy system with a spatial resolution of 0.6 μm [7]. Isikman *et al.* demonstrated a lens-free optical tomographic microscope with a large field of view [8]. Seo *et al.* demonstrated a lens-free holographic imaging system that can perform on-chip cytometry [9]. All these methods are based on an unconventional imaging technology called the lens-free imaging technology which is gaining in popularity because of its advantages such as high throughput, compact size, low cost, and reagent-free detection [10].

A lens-free imaging system is a simple arrangement of optoelectronic devices for capturing the diffraction patterns of micro-objects that are located very close to the optoelectronic sensor, e.g., a complementary metal–oxide–semiconductor (CMOS) or charge-coupled device (CCD) image sensor [11]. The characteristics of a diffraction pattern generated upon illumination of the sample plane with coherent light are governed by the physical and optical properties of the object, such as its size, shape, and refractive index [11,12]. These properties can be obtained by characterizing the captured diffraction pattern [12]. However, to date, these types of analysis have been done manually or using semi-automated software such as Image J, which requires manual assistance to provide the threshold range, circularity, and other parameters. Further, this type of software is generally designed for the analysis of optical micrographs. Therefore, there is a need for a fully automated, dedicated algorithm for fast, hassle-free characterization of the diffraction patterns in a lens-free image. In our previous work we tried to implement an automated method based on global threshold [13,14]. However, the algorithm shows a correlation in the range of 0.68 to 0.89 for microbeads. This is due to the variation in the background intensity within the same sample. We tried to address this issue by adopting a local threshold method.

In this paper, we introduce an automated method that can automatically detect, obtain, and analyze the features of the diffraction patterns of micro-objects. The approach is based on the detection of the midpoint of a diffraction pattern, followed by identification of the diffraction parameters, such as the central maxima value (CMV), width of the central maxima (WCX), width of the central minima (WCN), and peak-to-peak distance (PPD) [13,14]. For this purpose, we develop a mechanism to locally obtain the signal pixels and a clustering procedure to acquire the central positions of the diffraction patterns. The detected parameters are used for filtering as well as to obtain the physical properties of the micro-objects. The algorithm is optimized for fast, accurate performance by comparing the results with standard microscope results. Finally, the performance of this approach is investigated for various samples: microbeads, RBCs, and HeLa, MCF7, and HepG2 cells. The quantified results are compared with the results of a conventional standard method to evaluate the agreement between the two.

## 2. Material and Methods

### 2.1. Lens-Free Imaging

**Figure 1:**
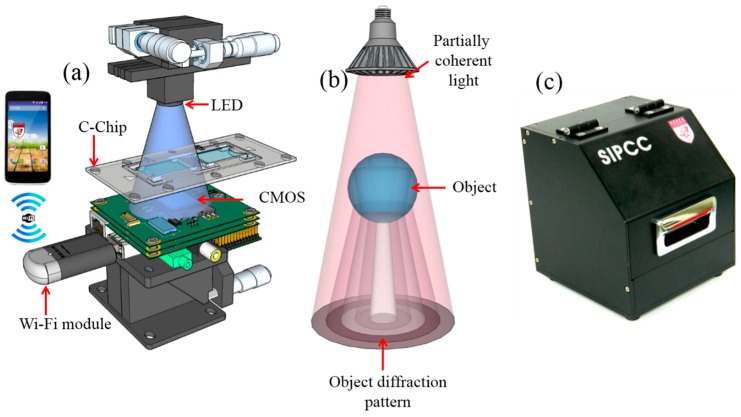
Schematic and working principle of the lens-free imaging system. (**a**) Schematic of the proposed system illustrating its simplicity with potential wireless file transfer facility; (**b**) cartoon of working principle of the formation of the diffraction pattern of a micro-object; (**c**) external view of the fabricated setup.

Cell imaging is among the basic methods used in cytometry and feature profiling of micro-objects. However, a high-resolution microscope is not the only choice for this purpose. The necessary vital information required for cell cytometry and particle analysis can be provided by alternative arrangements such as a lens-free shadow imaging system [6]. The lens-free imaging system is a compact system consisting of a CCD or CMOS image sensor and a partially coherent light source, e.g., a light-emitting diode (LED) [13,14,15]. A schematic of the custom-built lens-free imaging system is shown in Figure 1. The schematic illustrates the simplicity of the fabricated setup. In our system, we used a five-megapixel (1920 × 2560 pixel) monochrome CMOS image sensor (EO-5012M, Edmund Optics, Barrington, NJ, USA) that can be purchased at 14 USD per chip for the order of 2400 chips (MT9P031, Aptina, Phoenix, AZ, USA), with a sensing area of 23.52 mm^2^ and unit pixel size of 2.2 μm, and a blue LED with a peak wavelength of 470 ± 0.5 nm (HT-P318FCHU-ZZZZ, Harvatek, Hsinchu City, Taiwan; costing approximately 3 USD) as a light source. A pinhole 300 ± 5 μm in size was mounted on the top of the LED to achieve uniform semi-coherent illumination that illuminates samples loaded in a transparent cell-counting chamber made of polymethylmethacrylate (C-Chip, C10288, Invitrogen, Waltham, MA, USA). A single-board computer (Raspberry Pi, Raspberry Pi foundation, Caldecote, UK) costing approximately 40 USD was used to record the captured images and to transmit them wirelessly to a smartphone or a PC, where they were auto-processed using the custom-developed software. An Edimax (Edimax Technology Co. Ltd, Xinbei, Taiwan) 2.4 GHz wireless adapter costing about 10 USD was used to facilitate the Wi-Fi connection. All these components were packed within 9.3 × 9.0 × 9.0 cm^3^. This fabricated system was used to obtain whole-frame lens-free images that offer a field of view approximately 25 times that of a 100× optical microscope.

### 2.2. Sample Preparation

We used several types of cell lines and microbead samples for this study. The preparation methods of these samples are described below.

#### 2.2.1. Polystyrene Microbeads

We used polystyrene microbeads (Thermo Scientific, Waltham, MA, USA) to examine the performance of the algorithm for particle counting and size determination. We prepared four heterogeneous samples with a wide particle size range (5–30 μm) by mixing in different concentrations with de-ionized (DI) water. These samples were then examined under the lens-free system and a standard optical microscope using a C-Chip.

#### 2.2.2. RBCs

RBC samples were collected from Korea University Ansan Hospital under institutional review board approval in a tube treated with ethylenediaminetetraacetic acid. The samples were then diluted with Roswell Park Memorial Institute (RPMI-1640, Thermo Scientific) media and loaded in a C-Chip for analysis under the proposed lens-free system and a standard optical microscope.

#### 2.2.3. HepG2 Cells

HepG2 cell lines were derived from human liver tissue from the American Type Culture Collection (ATCC HB-8065, Manassas, VA, USA) and grown in a high-glucose growth medium (Dulbecco Modified Eagle Medium, DMEM) supplemented with 10% heat-inactivated fetal bovine serum, 0.1% gentamycin, and a 1% penicillin/streptomycin solution under 95% relative humidity and 5% CO_2_ at 37 °C. The cells were then trypsinized and separated from the 24-well plate and incubated for 2–5 min at 37 °C. The incubated cells were washed and diluted with DMEM solution and then loaded in a C-Chip for analysis under the lens-free system and optical microscope.

#### 2.2.4. MCF7 Cells

A human breast cancer cell line (MCF7) was obtained from the American Type Culture Collection (ATCC HTB-22, Manassas, VA, USA). The cells were maintained in a solution of DMEM containing 1% penicillin/streptomycin solution, 0.1% gentamycin, and 10% calf serum at 95% relative humidity and 5% CO_2_ at 37 °C. These cells were then trypsinized to separate them from the well plate, followed by incubation for 2–5 min at 37 °C. The cells were then washed with DMEM solution and loaded in a C-Chip for examination under the lens-free system and optical microscope.

#### 2.2.5. HeLa Cells

A human cervical cancer cell line (HeLa, ATCC CCL-2) was collected from the American Type Culture Collection (Manassas, VA, USA). The cells were then maintained in a solution of DMEM containing 1% penicillin/streptomycin solution, 0.1% gentamycin, and 10% calf serum at 95% relative humidity and 5% CO_2_ at 37 °C. The cells were separated from the 24-well plate by applying a trypsin solution and incubated for 2–5 min at 37 °C. After incubation, the cells were washed and diluted in a DMEM solution and loaded in C-Chip for examination under the lens-free system and optical microscope.

### 2.3. Algorithm

The algorithm used in this study contains a number of steps. The following sections describe the processes involved in this method in detail.

#### 2.3.1. Summary of the Algorithm

**Figure 2:**
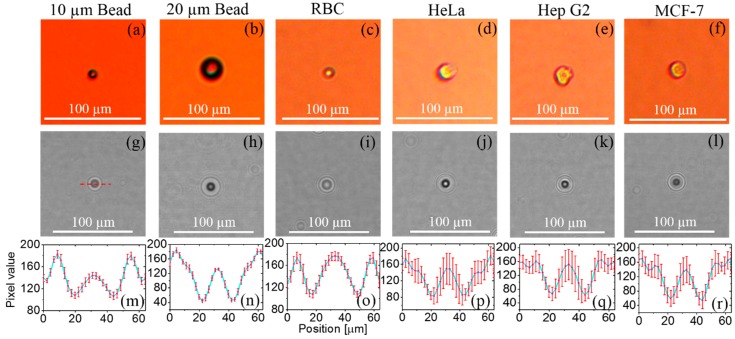
Diffraction pattern analysis. Optical micrographs of a single (**a**) 10 μm bead; (**b**) 20 μm bead; (**c**) RBC; (**d**) HeLa cell; (**e**) HepG2 cell; and (**f**) MCF7 cell; (**g**)–(**l**) diffraction images corresponding to (**a**)–(**f**), respectively, (**m**)–(**r**) intensity profiles corresponding to (**g**)–(**l**), with statistical differences of 10 samples from each of these cell lines.

**Figure 3:**
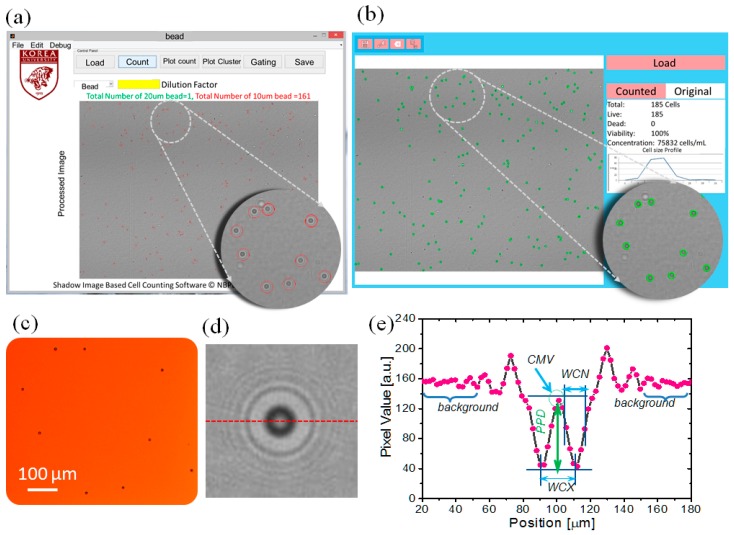
Snapshot of the custom-developed application software. (**a**) Snapshot of custom-developed Windows application software; (**b**) snapshot of custom-developed Android application; (**c**) optical micrograph of region of interest at 100× magnification; (**d**) diffraction pattern of a single microparticle; (**e**) intensity profile of the diffraction pattern in (**d**) explaining the custom-developed diffraction parameters along the dotted red line in (**d**).

As the signals of the diffraction patterns in a whole-frame lens-free image are significantly affected by that of the background, the signals can be filtered using a threshold [16]. However, owing to the large field of view and non-uniform background, a global threshold is unsuitable for this purpose (see the comparison of existing segmentation method ‘graythresh’ with our custom developed method in the Figures S1, S2 and Table S1 of the Supplementary Materials). Again, the strength of the signal and value of the background may vary from sample to sample. To understand this, we studied lens-free images of different samples (see Figure 2) and by evaluating the 3D intensity profile. This shows the variation in the background values and pixel values of the signals. In this context, it is necessary to acquire the signals locally [17]. We did this using a 10 × 10 patch wise technique method [18]. To locate the midpoint of the filtered diffraction pattern, we employed a clustering method using a 25 × 25 patch wise technique. The midpoints of the clusters were obtained by averaging the spatial coordinates of each of the filtered cluster elements in the 25 × 25 window. Finally, we filtered the unwanted diffraction noise by evaluating the circularity of the diffraction patterns. This algorithm is then implemented as application software as shown in the Figure 3. The following are the steps for the realization of this algorithm.

#### 2.3.2. Study of Diffraction Images

**Figure 4:**
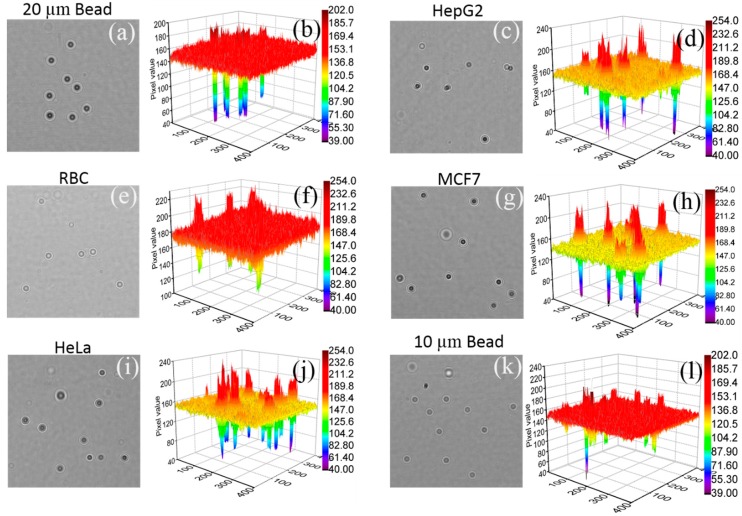
3D intensity plot of lens-free images of six samples. (**a**) 20 μm bead; (**c**) HepG2; (**e**) RBC; (**g**) MCF7; (**i**) HeLa cell; and (**k**) 10 μm bead. (**b**), (**d**), (**f**), (**h**), (**j**), and (**l**) are the 3D intensity plots corresponding to (**a**), (**c**), (**e**), (**g**), (**i**), and (**k**), respectively.

We conducted a study to understand the features of the diffraction patterns of the micro-objects that were captured by the fabricated lens-free imaging system. For this study, we selected six types of samples: 10 μm and 20 μm polystyrene beads, RBCs, and the HeLa, HepG2, and MCF7 cell lines. We ensured the type and size of the sample by taking the optical micrograph of the same sample in 100× optical zoom. We evaluated the intensity profile for 10 different diffraction patterns of each type of sample manually using Image J (NIH, Bethesda, MD, USA) [15]. The intensity profiles of all these samples are shown in Figure 2, which demonstrates that the diffraction patterns have almost the same features as the intensity profiles. However, the diffraction parameters for each type of sample differ significantly. We identified these parameters as the CMV, WCX, WCN, and PPD [13], as shown in Figure 3e. These parameters represent the physical optical properties of the micro-objects [14]. To detect and characterize these parameters, it is important to precisely locate the position of the diffraction pattern. We also studied the 3D intensity profile (see Figure 4 of the samples to understand the variation of the intensity profile within the sample as well as in between the samples.

#### 2.3.3. Signal Acquisition

**Figure 5:**
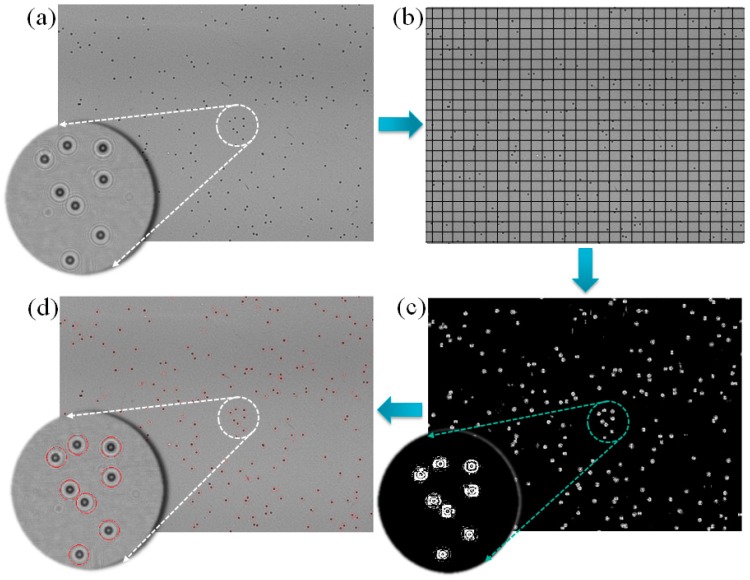
Schematic of the workflow of the algorithm. (**a**) Whole-frame image from the fabricated lens-free imaging system; (**b**) schematic representation (not the actual representation of 10 × 10 window) of the windowing method; (**c**) filtered binary image of (**b**); (**d**) detected and marked diffraction patterns in a whole-frame lens-free image.

The signals of the diffraction patterns in a whole-frame image from a lens-free system were filtered using a 10 × 10 sliding window filter. A schematic representation of the windowing is shown in Figure 5b. The threshold value in a 10 × 10 window was calculated by finding the difference between the maxima and minima of the pixels in that window. If the pixel value difference between the maxima and minima in that window exceeds 30 units, then the threshold value is equal to half the sum of the maxima and minima. Any pixel in that window having a value greater or less than the threshold value was recorded and stored in a binary image of the same size as the original image. The window was kept moving until it reached the end of the image.

#### 2.3.4. Clustering

The filtered binary image contains only the probable signal pixels with their actual spatial coordinates. Figure 5c shows a filtered binary version of a lens-free image. However, these binary images are sparse in nature. The pixels in a filtered binary image are not continuous, and edge pixels from two neighboring diffraction patterns are very difficult to assign to a particular origin (see the region of interest in Figure 5c). The image also contains some unwanted noise pixels. To eliminate these shortcomings, a clustering method was employed. A 25 × 25 patch-wise technique was used to find the spatial distance between the signal pixels. The pixels inside a particular window were grouped together on the basis of the calculated spatial distance. Any pixel having a spatial distance less than 3 pixel units from the neighboring pixels was considered as a probable cluster member, and any isolated pixels were neglected. The window was kept moving until it reached the end of the image. The tentative midpoint of a cluster or diffraction pattern was calculated by averaging the spatial coordinates of each cluster element.

#### 2.3.5. Diffraction Parameter Acquisition

The CMV of a diffraction pattern is the pixel value of the midpoint. The CMVs of the diffraction patterns in a whole-frame lens-free image were obtained by mapping the calculated midpoint positions from the binary clusters to the original image. The WCX of a diffraction pattern was obtained across the midpoint by calculating the number of all pixels in a row (or column), where the pixel values were similar to the CMV. The WCN of a diffraction pattern was obtained across the midpoint by calculating the number of all pixels in a row (or column), where the pixel values were less than the CMV.

#### 2.3.6. Circularity Filter

A study of the diffraction images of six different types of samples reveals that the diffraction patterns are circular. This property is an advantage for filtering unwanted diffraction patterns. Therefore, we implemented another filter, which evaluates the circularity of the diffraction patterns by calculating the aspect ratio of the WCX and the WCN. If the aspect ratio (WCX vertical/WCX horizontal) of a particular pattern is equal to 1, then it was considered; otherwise, it was neglected. Similarly, the circularity was obtained using the WCN. The filtered diffraction patterns were considered final and marked (Figure 5d).

#### 2.3.7. Size Determination

In this step, the PPDs of the filtered diffraction patterns were calculated. The PPD was evaluated by calculating the difference between the CMV and the minima of the diffraction pattern. The concentrations of the microparticles were calculated by evaluating the total number of filtered diffraction patterns. The sizes of the microparticles were obtained by converting the PPD to the original image as described in our previous work [14]. In this method, the original size of the objects from the detected PPD value were obtained using the equation

**Figure 6:**
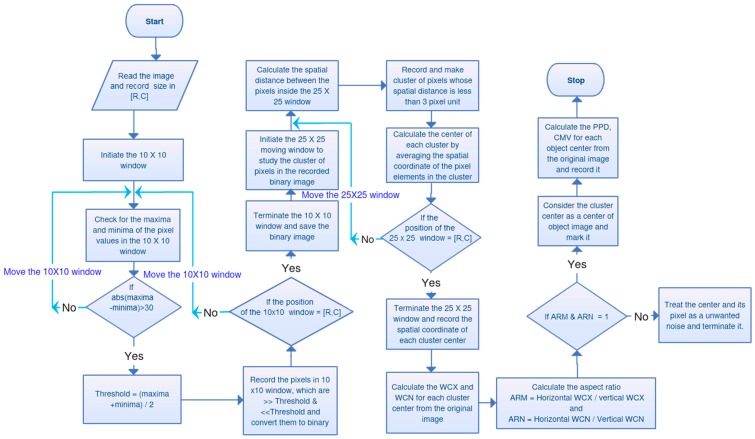
Concise flowchart of the algorithm.

*Y* = 0.28*X* (1)

where *Y* is the original size, and *X* is the PPD. A concise flowchart of all the steps of the algorithm is depicted in Figure 6.

## 3. Results and Discussion

As described in an earlier section, the features of the diffraction patterns of micro-objects were evaluated for six different types of samples. For this purpose, we used a 10 μm bead, a 20 μm bead, an RBC, and single HeLa, HepG2, and MCF7 cells. Optical micrographs and the corresponding lens-free images of each sample are shown in Figure 2. The intensity profiles were obtained manually by selecting an array of signal pixels from the diffraction patterns (see the red line in Figure 2g) using Image J (NIH, USA) image processing software. Thus, we obtained 10 different profiles of each type of sample for statistical study. The average coefficients of variation of the 10 μm bead, 20 μm bead, RBC, and HeLa, HepG2, and MCF7 cells are 0.53, 0.08, 0.06, 0.19, 0.23, and 0.21, respectively. The features of the diffraction patterns of all six samples are almost identical. However, each sample has different diffraction parameters. The statistical averages, *i.e.*, the average of the diffraction parameters of 10 samples, for the 10 μm bead, 20 μm bead, RBC, and HeLa, HepG2 and MCF7 cells are as shown in the Table 1. This shows that the physical sizes of the diffraction patterns are almost the same, as there are very few differences in the WCX and WCN for all the sample types. However, the PPD and CMV differ significantly for each type of cell.

We also studied the variation in the pixel values, particularly that of the background of the lens-free image, for each type of sample. We used the lens-free images of the 10 µm bead, 20 µm bead, RBC, HeLa, HepG2, and MCF7 cells. The 3D profiles of these images are shown in Figure 4. The study shows that the background of the lens-free image varies with the sample type. This is especially noticeable in the RBC sample (about 180) in Figure 4f, compared to other samples (approximately 160). This is due to the opacity, refractive index, and cell density of the samples. Again, in some samples (Figure 4f,l) the signal intensity is comparatively low (see magnified figures in the Supplementary Figure S3).

**Figure 7:**
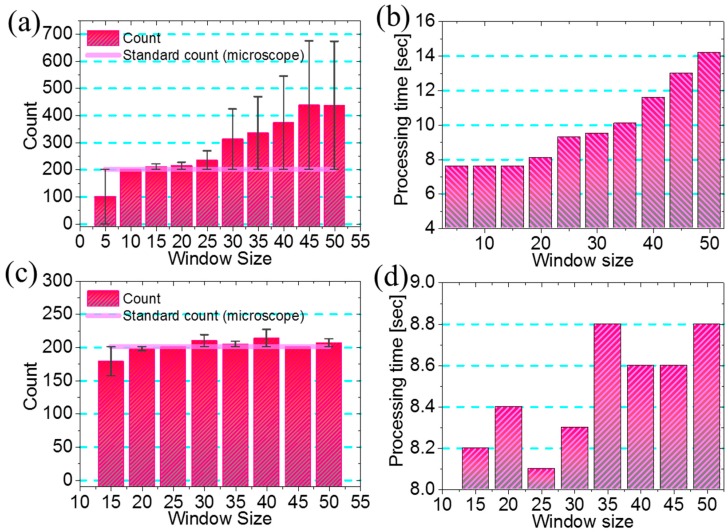
Optimization of the windowing method. (**a**) Window size *vs.* counting result for obtaining the local threshold; (**b**) window size *vs.* processing time for obtaining the local threshold; (**c**) window size *vs.* counting result for clustering of filtered binary image; (**d**) window size *vs.* processing time for clustering of filtered binary image.

To overcome all of these shortcomings, we need a mechanism to obtain the adaptive threshold. Therefore, we implemented the patch-wise technique method for this purpose. To determine the appropriate window size, we tested the algorithm with different window sizes and compared the results with the standard result. We also evaluated the processing time required for each window size. The results are shown in Figure 7a,b, respectively. The result indicates that the 10 × 10 window, which provides more accurate results in less time, is ideal for obtaining the local threshold. This is also indicated by the size of the diffraction patterns. The total width (WCX + WCN) of the diffraction pattern for each sample is approximately 10–15 pixel units. Therefore, we implement the 10 × 10 window in this algorithm.

Again, the filtered binary images are sparse in nature (Figure 5c). To determine the midpoint of the diffraction patterns, we need to find the actual group of sparse signal pixels. For this purpose, we developed a clustering method in which the spatial distance between the signals is calculated. This was done by implementing a patch-wise technique to locally compute the spatial distances. We also optimized the size of this window for better performance in less processing time. The results are shown in Figure 7c,d. The results indicate that the 25 × 25 window exhibits better performance with less processing time. We used these optimized parameters in the algorithm and implemented it on the custom-developed Android and Windows applications. Snapshots of the application layout are shown in Figure 3a,b. Using this application software, we evaluated the counting and size profiling of the six types of samples.

**Figure 8:**
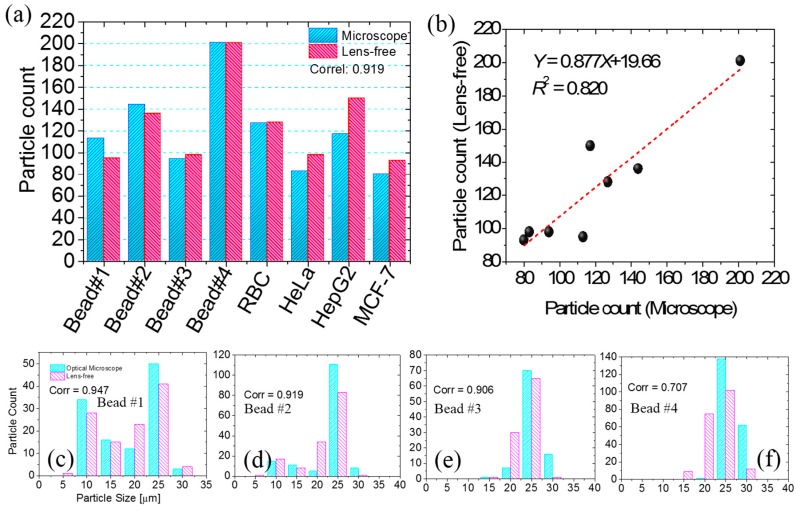
Comparison of automatically processed result with standard microscope results. (**a**) Comparison for six different samples; (**b**) linearity comparison of the automatically processed and standard microscope results for six samples in (**a**); (**c**)–(**f**) comparison of auto-processed size results with the standard microscope results for bead samples #1–4.

The counting results from the custom-developed algorithm for all six samples were compared with the standard optical microscope results. Figure 8a compares the counting performance of the two modalities. The comparison shows a correlation of 0.91. The linearity of the counting results from the two modalities is compared in Figure 8b. This shows linear behavior with a slope of 0.877 and *R*^2^ value of 0.820. In addition, the size profiling results from the automated method were compared with the results from the standard optical microscope. The results for bead samples #1–4 are shown in Figure 8c–f, respectively. The correlation coefficients of these comparisons are 0.947, 0.919, 0.906, and 0.707, respectively, which indicates that the results of the proposed system agree with those of the conventional method. The average error for the size determination is about 1.6 µm (see Table S2 in Supplementary).

## 4. Conclusions

In summary, an automated micro-object counting method for a lens-free imaging system was demonstrated. A comparison of the results obtained using this approach with those obtained using the standard method shows good agreement between the two modalities. The correlation coefficient of 0.91 and slope of 0.877 show the agreement and linearity between the automated and conventional approaches. Further, a comparison of the size results shows correlations of 0.70 or greater, which indicates the feasibility of automated size characterization using the lens-free system. The lens-free system is made of inexpensive components, e.g., an LED costing 3 USD and a CMOS image sensor costing 14 USD, the cost of which is negligible compared to that of a conventional auto-detection system. The automated algorithm processes the result within the range of 15 to 20 s. Therefore, this would be a cost-effective option for many research facilities. This type of system, along with the dedicated algorithm, can evaluate several hundred diffraction patterns of micro-objects including RBCs and HeLa, HepG2, and MCF7 cells in a few minutes using a moderate smart phone. This combination of a Wi-Fi-enabled lens-free system and automated detection software would provide a cost-effective telemedicine facility for early diagnosis in resource-limited settings. However there is more scope to upgrade the system to analyze the poor signals from small microparticles (less than 2 µm). This may be achieved by introducing more sophisticated sensors with lower pixel size alongside high density. An automated feature recognition algorithm such as a deep learning algorithm may be an added advantage for auto recognition of the type of cells. This will eradicate the dependency of the current algorithm on the diffraction parameters, which may be a scope for future research.

## Figures and Tables

**Table 1:** Statistical average of 10 samples for the diffraction parameters.

Sample/Diffractin Parameters	10 μm Bead	20 μm Bead	RBC	HeLa	HepG2	MCF7
CMV	144	132	176	149	151	139
WCX	8	7	11	6	7	7
WCN	4	4	5	4	5	4
PPD	38	86	69	65	89	85

## Supplementary Materials

The Supplementary files are available online at www.mdpi.com/2075-4418/6/2/17/s1.
