# Supplementary material for: Automated Micro-Object Detection for Mobile Diagnostics Using Lens-Free Imaging Technology
Source: Diagnostics (Basel). 2016 May 5;6(2):17. doi: 10.3390/diagnostics6020017 (PMC4931412; doi:10.3390/diagnostics6020017)
Supplement: Supplementary file 1 [file diagnostics-06-00017-s001.pdf]

# Supplementary Materials: Automated Micro-Object Detection for Mobile Diagnostics Using Lens-Free Imaging Technology

**Mohendra Roy, Dongmin Seo, Sangwoo Oh, Younghun Chae, Myung-Hyun Nam and Sungkyu Seo**

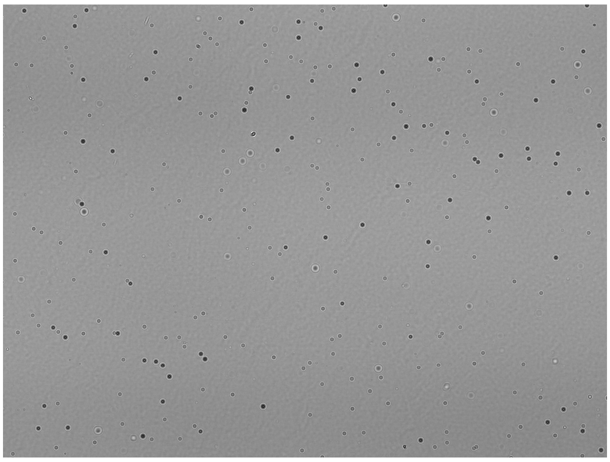

(**a**)

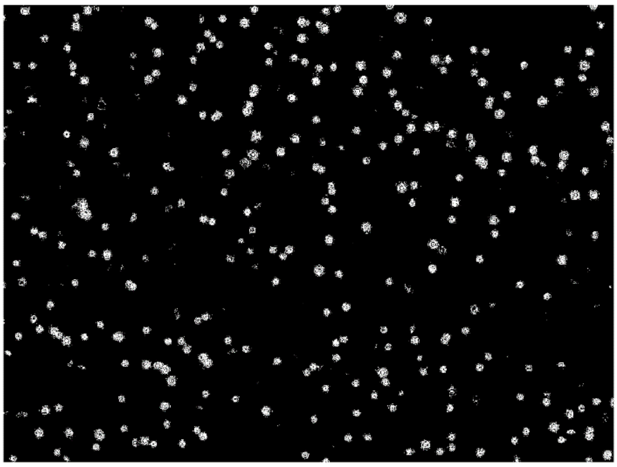

(**b**)

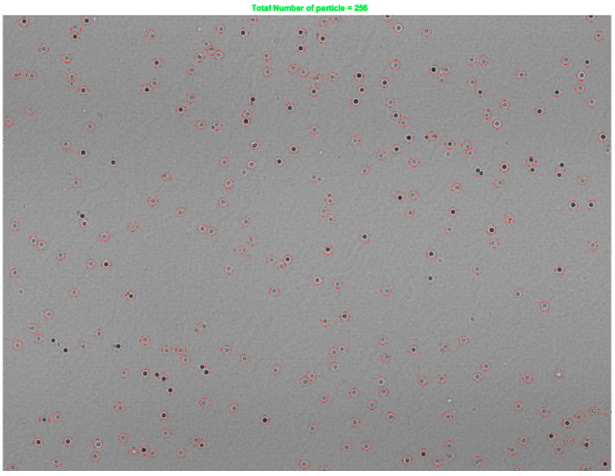


(**c**)

**Figure S1.** Processed by custom developed algorithm, (**a**) Original Image; (**b**) Binary image after threshold; (**c**) Counted and marked.

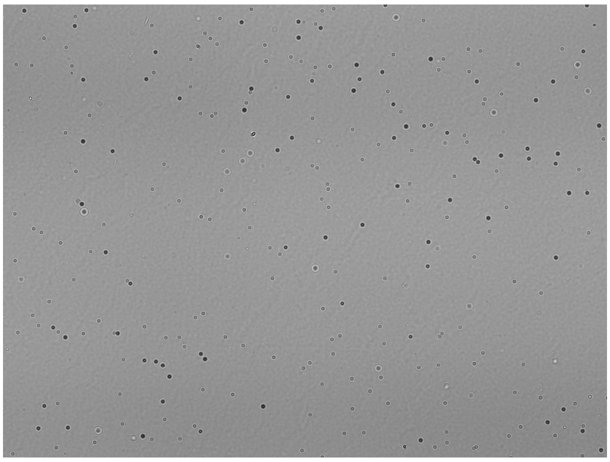

(**a**)

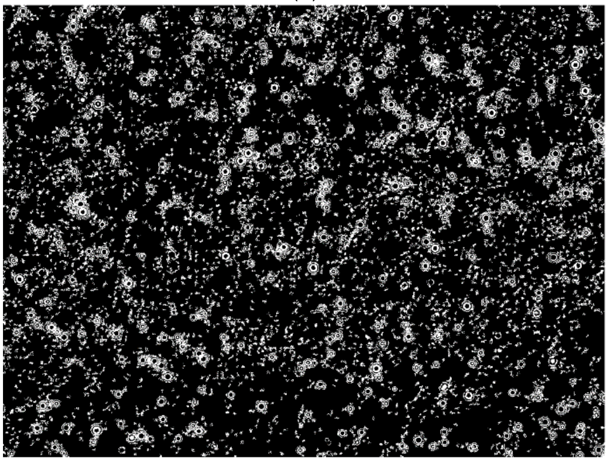

(**b**)

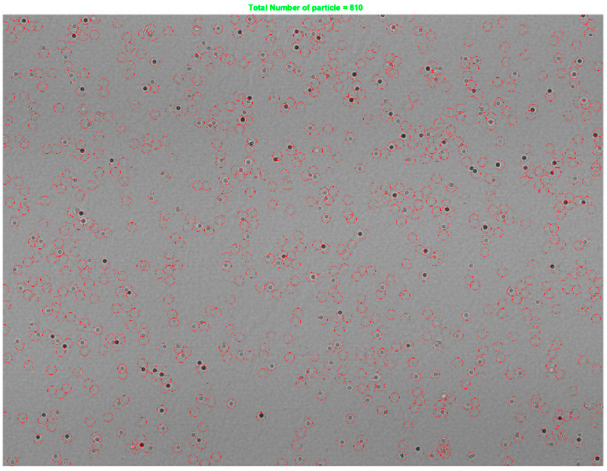


(**c**)

**Figure S2.** Processed by existing algorithm (graythresh), (**a**) Original Image; (**b**) Binary image after threshold; (**c**) Counted and marked. (http://www.mathworks.com/help/images/image-enhancement-and-analysis.html?s_tid=gn_loc_drop&refresh=true).

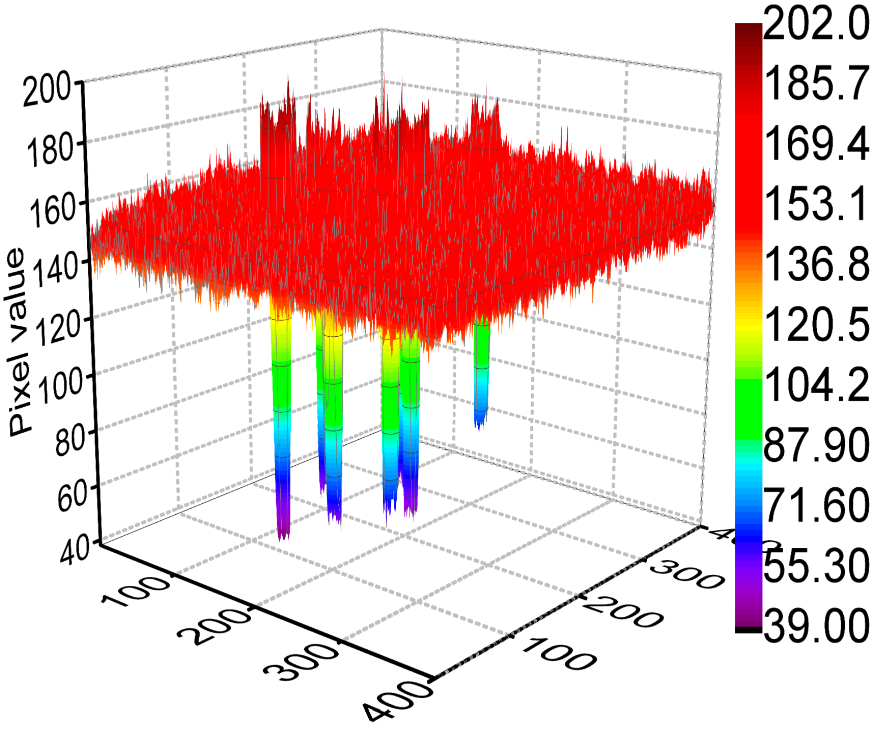
Pixel value
200
180
160
140
120
100
80
60
40
100
200
300
400
100
200
300
202.0
185.7
169.4
153.1
136.8
120.5
104.2
87.90
71.60
55.30
39.00

(**a**)

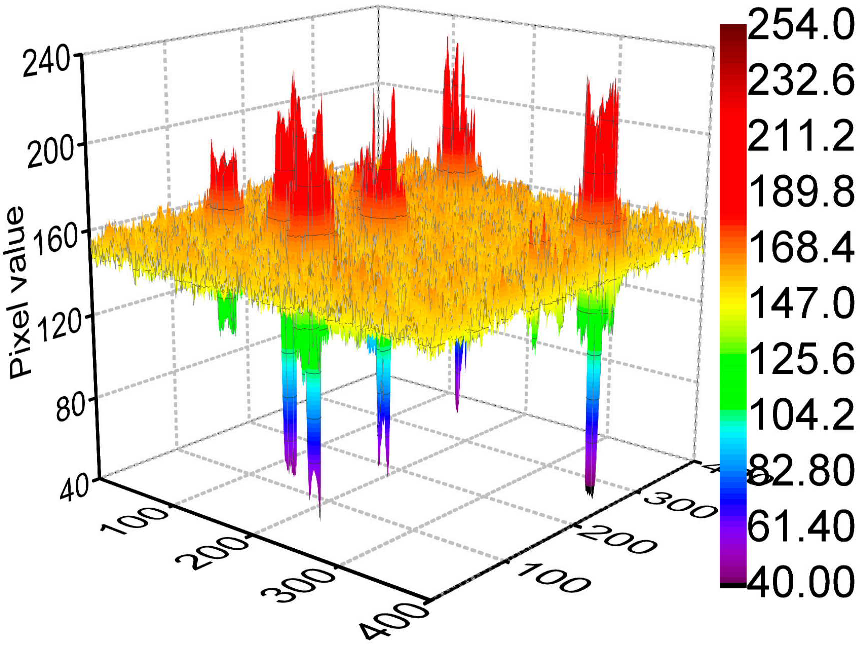
Pixel value
240
200
160
120
80
40
100
200
300
400
100
200
300
254.0
232.6
211.2
189.8
168.4
147.0
125.6
104.2
82.80
61.40
40.00

(**b**)

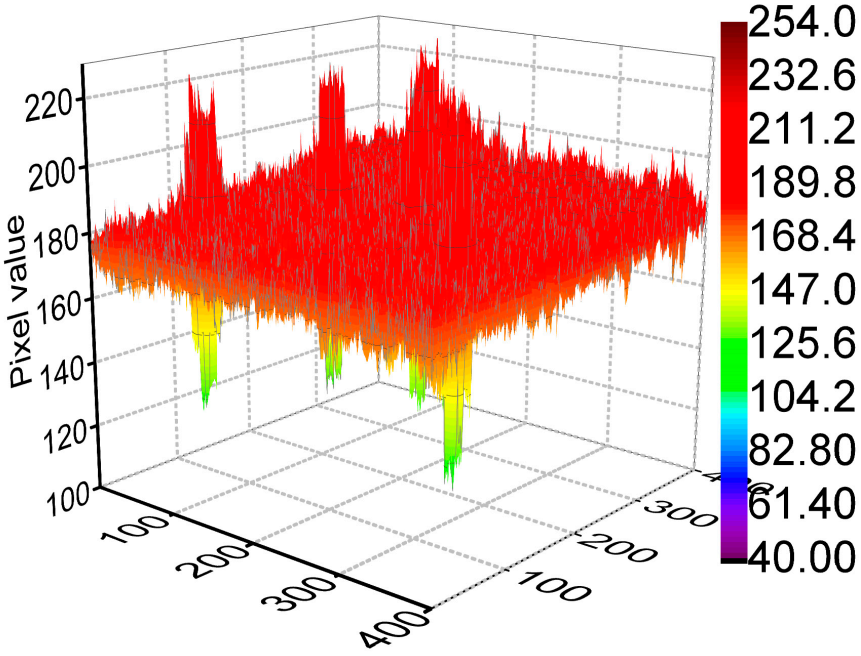
Pixel value
220
200
180
160
140
120
100
100
200
300
400
100
200
300
254.0
232.6
211.2
189.8
168.4
147.0
125.6
104.2
82.80
61.40
40.00

(**c**)

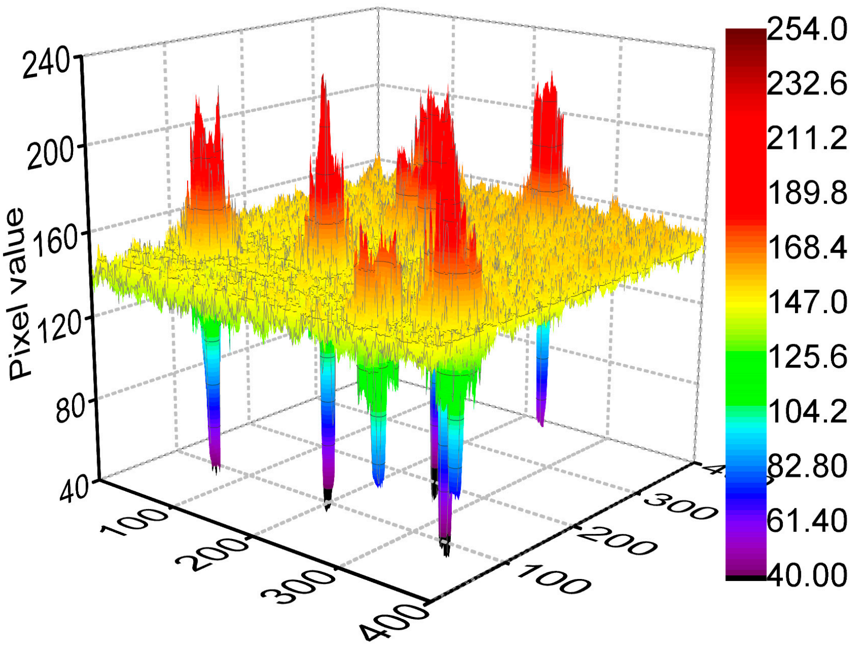
Pixel value
240
200
160
120
80
40
100
200
300
400
100
200
300
254.0
232.6
211.2
189.8
168.4
147.0
125.6
104.2
82.80
61.40
40.00

(**d**)

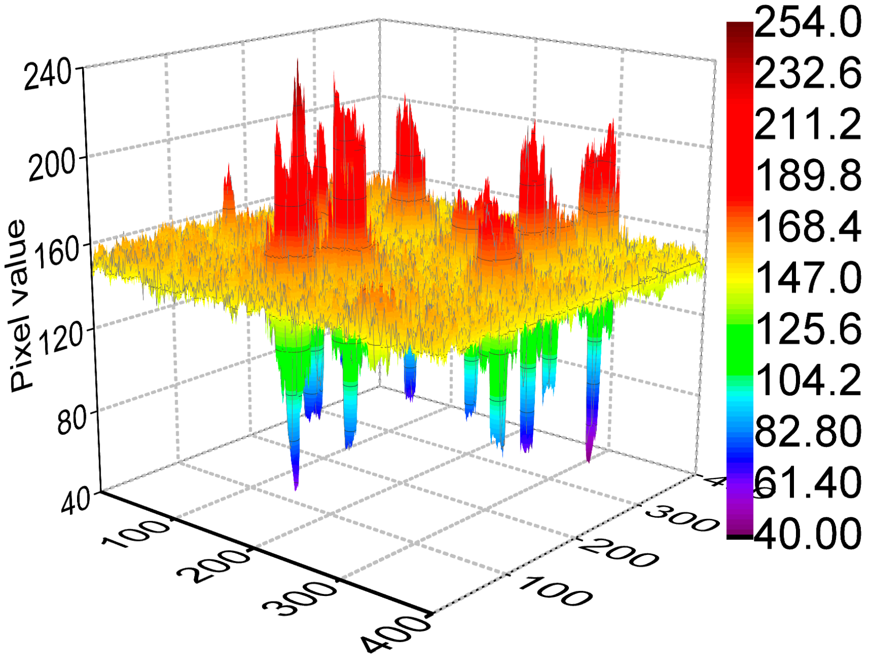


(**e**)

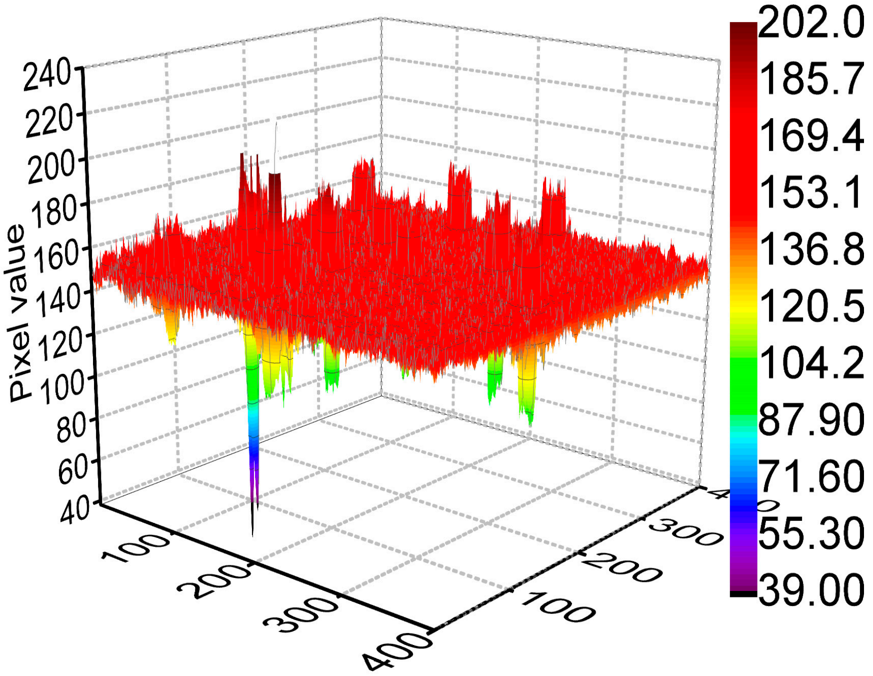


(**f**)

**Figure S3**. 3D intensity plot of (**a**) 20 µm bead; (**b**) HepG2; (**c**) RBC; (**d**) MCF7; (**e**) HeLa; (**f**) 10 µm bead.

**Table S1.** Comparison of automated methods for the counting of lens-free images of microbeads.

| Methods | Manual Counting | Existing Algorithm (Graythresh) | Custom Algorithm |
|---|---|---|---|
| Count | 249 | 810 | 256 |

**Table S2.** Comparison of actual size of microparticle by microscope vs. size by automated algorithm.

| Sample No | Actual Size by Microscope (Micrometer) | Size Determine by Algorithm (Micrometer) | Difference (Micrometer) |
|---|---|---|---|
| 1 | 20.2 | 20 | 0.2 |
| 2 | 21.35 | 21.71428571 | 0.364285714 |
| 3 | 12.55 | 12 | 0.55 |
| 4 | 24.69 | 22.85714286 | 1.832857143 |
| 5 | 24.69 | 23.42857143 | 1.261428571 |
| 6 | 11.78 | 10.57142857 | 1.208571429 |
| 7 | 11.78 | 9.142857143 | 2.637142857 |
| 8 | 11.2 | 10.57142857 | 0.628571429 |
| 9 | 10.1 | 12 | 1.9 |
| 10 | 11.22 | 10.85714286 | 0.362857143 |
| 11 | 10.1 | 8.857142857 | 1.242857143 |
| 12 | 10.66 | 10 | 0.66 |
| 13 | 23.57 | 21.71428571 | 1.855714286 |
| 14 | 11.78 | 11.42857143 | 0.351428571 |
| 15 | 10.1 | 8 | 2.1 |
| 16 | 13.47 | 11.42857143 | 2.041428571 |
| 17 | 12.35 | 10 | 2.35 |
| 18 | 12.91 | 9.428571429 | 3.481428571 |
| 19 | 24.64 | 23.71428571 | 0.925714286 |
| 20 | 19.64 | 20.57142857 | 0.931428571 |
| 21 | 12.35 | 9.714285714 | 2.635714286 |
| 22 | 11.78 | 10.28571429 | 1.494285714 |
| 23 | 10.1 | 7.428571429 | 2.671428571 |
| 24 | 11.22 | 7.428571429 | 3.791428571 |
| 25 | 23.1 | 22.28571429 | 0.814285714 |
| 26 | 10.66 | 8.571428571 | 2.088571429 |
| 27 | 26.37 | 22.28571429 | 4.084285714 |
| 28 | 10.66 | 9.428571429 | 1.231428571 |
| 29 | 10.1 | 10.85714286 | 0.757142857 |
| 30 | 11.78 | 11.42857143 | 0.351428571 |
| 31 | 10.66 | 12.57142857 | 1.911428571 |
| 32 | 20.76 | 24 | 3.24 |
| 33 | 10.1 | 8.857142857 | 1.242857143 |
| 34 | 18.52 | 21.71428571 | 3.194285714 |
| 35 | 20.2 | 22 | 1.8 |
| 36 | 10.1 | 10.28571429 | 0.185714286 |
| 37 | 21.32 | 24.57142857 | 3.251428571 |
| 38 | 11.22 | 13.71428571 | 2.494285714 |
| 39 | 22.45 | 23.71428571 | 1.264285714 |
| 40 | 25.25 | 25.71428571 | 0.464285714 |
| 41 | 24.13 | 23.71428571 | 0.415714286 |
| 42 | 22.45 | 23.42857143 | 0.978571429 |
| 43 | 21.32 | 21.14285714 | 0.177142857 |
| 44 | 10.66 | 10 | 0.66 |
| 45 | 10.66 | 12 | 1.34 |
| 46 | 10.66 | 11.42857143 | 0.768571429 |
| 47 | 22.45 | 22 | 0.45 |
| 48 | 14.4 | 12.57142857 | 1.828571429 |
| 49 | 10.66 | 8.571428571 | 2.088571429 |
| 50 | 10.1 | 8.857142857 | 1.242857143 |
| 51 | 12.35 | 10.57142857 | 1.778571429 |
| 52 | 10.66 | 11.14285714 | 0.482857143 |
| 53 | 23.01 | 22.28571429 | 0.724285714 |
| 54 | 25.25 | 24 | 1.25 |

| | | | |
|---|---|---|---|
| 55 | 12.35 | 11.14285714 | 1.207142857 |
| 56 | 11.78 | 10.28571429 | 1.494285714 |
| 57 | 12.35 | 13.42857143 | 1.078571429 |
| 58 | 11.22 | 12 | 0.78 |
| 59 | 10.1 | 11.42857143 | 1.328571429 |
| 60 | 11.78 | 11.42857143 | 0.351428571 |
| 61 | 12.35 | 12.28571429 | 0.064285714 |
| 62 | 19.08 | 22.28571429 | 3.205714286 |
| 63 | 10.66 | 8.857142857 | 1.802857143 |
| 64 | 10.66 | 11.42857143 | 0.768571429 |
| 65 | 10.1 | 9.142857143 | 0.957142857 |
| 66 | 10.66 | 8.285714286 | 2.374285714 |
| 67 | 10.1 | 11.42857143 | 1.328571429 |
| 68 | 11.78 | 10.85714286 | 0.922857143 |
| 69 | 10.1 | 7.142857143 | 2.957142857 |
| 70 | 19.08 | 21.14285714 | 2.062857143 |
| 71 | 10.1 | 10 | 0.1 |
| 72 | 10.1 | 9.142857143 | 0.957142857 |
| 73 | 12.91 | 10.57142857 | 2.338571429 |
| 74 | 11.22 | 10.28571429 | 0.934285714 |
| 75 | 10.66 | 9.142857143 | 1.517142857 |
| 76 | 10.1 | 11.71428571 | 1.614285714 |
| 77 | 11.78 | 11.14285714 | 0.637142857 |
| 78 | 12.35 | 8.857142857 | 3.492857143 |
| 79 | 24.13 | 23.71428571 | 0.415714286 |
| 80 | 10.66 | 8.571428571 | 2.088571429 |
| 81 | 20.76 | 24 | 3.24 |
| 82 | 24.13 | 25.14285714 | 1.012857143 |
| 83 | 10.66 | 13.14285714 | 2.482857143 |
| 84 | 24.13 | 24.85714286 | 0.727142857 |
| 85 | 10.1 | 10.28571429 | 0.185714286 |
| 86 | 10.66 | 11.42857143 | 0.768571429 |
| 87 | 10.66 | 9.428571429 | 1.231428571 |
| 88 | 20.76 | 22 | 1.24 |
| 89 | 23.01 | 24.57142857 | 1.561428571 |
| 90 | 12.35 | 10.85714286 | 1.492857143 |
| 91 | 10.66 | 8.857142857 | 1.802857143 |
| 92 | 22.45 | 24.85714286 | 2.407142857 |
| 93 | 11.78 | 9.714285714 | 2.065714286 |
| 94 | 12.35 | 12.85714286 | 0.507142857 |
| 95 | 21.89 | 23.14285714 | 1.252857143 |
| 96 | 10.66 | 9.428571429 | 1.231428571 |
| 97 | 11.22 | 10 | 1.22 |
| 98 | 10.1 | 9.428571429 | 0.671428571 |
| 99 | 20.76 | 23.14285714 | 2.382857143 |
| | | **average Difference** | **1.647908623** |
